# *Cannabis sativa*: From Plants to Humans

**DOI:** 10.3390/ijms252413288

**Published:** 2024-12-11

**Authors:** Daniela Trono

**Affiliations:** Research Centre for Cereal and Industrial Crops, Council for Agricultural Research and Economics (CREA), S.S. 673, Meters 25200, 71122 Foggia, Italy; daniela.trono@crea.gov.it

This Special Issue aims to highlight some of the most recent developments in the study of *Cannabis sativa* by collecting contributions that range from studies on the crop and its interaction with the environment and pathogens to the pharmaceutical applications of cannabinoid-based drugs, also including the health risks associated with the consumption of *C. sativa* psychoactive derivatives.

*C. sativa* is an herbaceous annual plant of the Cannabaceae family that was domesticated in Central Asia about 12,000 years ago and progressively spread worldwide [1]. Five different chemotypes of *C. sativa* can be distinguished based on the levels of Δ^9^-tetrahydrocannabinol (THC), cannabidiol (CBD), and cannabigerol (CBG): chemotype I with high (>0.3%) THC and low CBD (<0.5%) levels, also referred to as the drug type or cannabis or marijuana; chemotype II with high (>0.3%) THC and high (>0.5%) CBD levels, also known as the intermediate type; and three chemotypes characterized by a low (<0.3%) level of THC, also known as ‘hemp’ or ‘industrial hemp’, which include chemotype III with a high (>0.5%) level of CBD, chemotype IV with a high (>0.3%) level of CBG, and chemotype V with almost undetectable cannabinoid levels [2,3].

While THC has psychoactive effects, the other cannabinoids are not psychoactive and may have beneficial effects for human health. Both these negative and positive effects are due to the interaction of these compounds with the human endocannabinoid system (EC), a signaling system that includes ligands, enzymes, and receptors, which influences various physiological processes throughout the human body [4].

The great interest that revolves around hemp and cannabis cultivation is linked to the multiple industrial applications of their derivatives. Regarding hemp, its stem fiber has long been used to make paper, textiles, and ropes [5] (Figure 1). More recently, it has also been used as reinforcement in construction materials and bioplastic production, whereas its biomass is used as a feedstock to make solid biofuels, ethanol, and biogas [5] (Figure 1). Additionally, hempseeds are used as ingredients in human and animal food as they are a valuable source of proteins, omega-6 and omega-3 fatty acids, antioxidants, and minerals [6] (Figure 1). Recently, hemp has also been receiving a great deal of attention for the high levels of phytochemicals that characterize its inflorescences. These are mainly represented by non-psychoactive cannabinoids, terpenes, and flavonoids, which have recognized positive effects for human health and are, therefore, used for the production of dietary supplements and cosmetic ingredients [7] (Figure 1).

As a result, the number of studies aiming to describe the chemical profile of hemp inflorescences has increased dramatically. In this context, Beleggia and coworkers (Contribution 1) analyzed the phytochemical profile of the inflorescences of both monoecious and dioecious hemp genotypes grown over three cropping years to assess the effect of genotype, year, and their interaction on the accumulation of cannabinoids, terpenes, phenolic compounds, and other less represented bioactive compounds. The authors reported that the effect of year prevailed as a source of variation in the accumulation of all the metabolites detected, although differences were observed between dioecious and monoecious genotypes. Indeed, the dioecious genotypes presented a more constant performance over the three years compared to the monoecious genotypes, with the highest and most stable phytochemical content. In particular, dioecious genotypes presented the highest levels of CBD and the sesquiterpenes α-humulene and β-caryophyllene, which may confer to their inflorescences an added value due to the important pharmacological properties of these compounds.

Regarding cannabis, its cultivation is linked to its medicinal and recreational uses. All pharmaceutical products that contain cannabinoids are referred to as cannabis-based products for medicinal use (CBPMs). CBPMs are used for the treatment of epileptic seizures, muscle spasticity in patients with multiple sclerosis or Parkinson’s disease, chemotherapy-induced nausea and vomiting, and pain, including that caused by inflammation [8] (Figure 2). In addition, a number of research and preclinical studies on in vivo models have proven their efficacy in the treatment of glaucoma, anxiety, and different types of cancer [9] (Figure 2). In this context, Seifalian and coworkers, in their review (Contribution 2), gave an overview of the composition and the different formulations of CBPMs and the guidelines for their use in the treatment of various diseases. Then, they focused on the mechanism of action of CBPMs in relation to pain relief and summarized all the clinical trials relating to CBPMs and pain management, highlighting that of the 27 clinical studies carried out to date, two-thirds have reported a significant beneficial effect in the majority of patients. Finally, they focused on the application of CBPMs to manage dysmenorrhea, a problem that affects up to 90% of women of reproductive age and that consists of painful menstrual cramping. The authors highlighted that the studies carried out to date are very scarce and only one trial is currently underway. Conversely, an increasing number of online start-ups offer CBD-based products in different formulations (e.g., edible form or tampon) without a direct claim for the treatment of dysmenorrhea, but indirectly insinuating the advantages of CBD in alleviating the pain experienced during the menstrual cycle. The authors concluded that a deeper comprehension of the mechanisms of action and safety profile of CBPMs is required for their effective use in treating dysmenorrhea.

The study by Tortolani and coworkers (Contribution 3) covered the ability of cannabinoids to exert an anti-inflammatory action on human tissues. In particular, the authors used an experimental model of inflamed human keratinocytes (HaCaT cells) to assess the ability of some rare cannabinoids, CBG, cannabigerolic acid (CBGA), cannabichromene (CBC), and Δ^9^-tetrahydrocannabivarin (THCV), to reduce inflammatory processes that are involved in several skin diseases, such as psoriasis, atopic dermatitis, and itch, as well as in innate and adaptive immunity. In this regard, it is known that the major elements of the EC system are produced in different cells of the skin, thus suggesting that they are involved in the pathophysiology of this tissue. The authors observed that the rare cannabinoids significantly blocked the inflammation through the inhibition of the release of pro-inflammatory interleukins (ILs), with a marked effect observed for CBGA and THCV. Moreover, these two cannabinoids were the only ones that determined a marked reduction in the expression of IL-31, which is involved in the itch sensation typical of psoriasis. The authors reported evidence that CBGA and THCV acted through the modulation of the TRPV1 receptor, which is known to be involved in psoriasis and contact dermatitis [10,11], and in combination with the endocannabinoid hydrolases to activate different proteins along the anti-inflammatory MAPK signaling pathway. Overall, these findings open the possibility for new treatments for inflammatory skin disorders.

As said above, evidence exists that cannabinoids exhibit anticancer activity and that this occurs through the reduction in cell viability, the induction of apoptosis, the impairment of angiogenesis, and the inhibition of invasion and metastasis [12]. In this context, Zeppa and coworkers (Contribution 4) investigated the anticancer effect of CBG in two human pancreatic ductal adenocarcinoma (PDAC) cell lines, PANC-1 and MIAPaCa-2, in order to analyze its interactions with epidermal growth factor receptor (EGFR)-mediated signaling pathways, which are known to play a pivotal role in tumor progression via proliferation, survival, invasion, and chemoresistance [13]. The authors observed that, in both cell lines, CBG reduced cell viability and induced autophagy and apoptosis through a downregulation of the RAS/RAF/MEK and Akt/mTOR pathways, two EGFR-mediated pathways involved in autophagy and apoptosis regulation [14]. In addition, they reported that the combinations of CBG with paclitaxel and gemcitabine, two drugs used together for treatment of advanced PDAC, resulted in greater cytotoxicity when compared to paclitaxel and gemcitabine alone in both cell lines.

While CBPMs have proven beneficial effects on human health, recreational cannabis consumption can, however, lead to a series of side-effects, which include cognitive alterations, impairment of perceptual–motor coordination, respiratory and cardiovascular diseases, and infertility [15] (Figure 2). Another serious health issue arises from cannabis usage during pregnancy and lactation, as it can be harmful to both the mother and her offspring [16] (Figure 2). In this context, Navarro and coworkers (Contribution 5) used for the first time an animal model to investigate the effect of exposure to dronabinol (a synthetic form of THC) during gestation and lactation on the emotional and cognitive aspects of offspring. The authors observed significant anxiogenic and depressive-like behaviors, as well as cognitive impairment, in male and female mice exposed pre- and postnatally to dronabinol. These altered behaviors were found to be associated with changes in the expression levels of genes within the hypothalamic–pituitary–adrenal axis and alteration in the development of the cerebral cortex and hippocampus. Dronabinol exposure also led to the disruption of the reward system, which in turn determined an increased alcohol consumption.

The renewed interest in industrial hemp applications and the increase in the use of cannabis for medicinal and recreational purposes has led to an increase in the cultivation of these crops [1,17]. Unfortunately, this has increased the number of pathogens that have affected this crop in recent years, the majority of which are represented by fungi and oomycetes, followed by viruses and viroids [18]. In this context, Punja and coworkers (Contribution 6) carried out a study to demonstrate how polymerase chain reaction (PCR)-based diagnostic approaches, namely, RT-PCR, multiplex RT-PCR, RT-qPCR, and ddPCR, as well as whole-genome sequencing (NGS) approaches, can be used to provide rapid and sensitive identification of pathogens on cannabis and hemp plants. By using these PCR-based assays, the authors detected those pathogens that represent the most significant economic concern for cannabis and hemp growers. *Fusarium* and *Pythium* spp. were detected on the roots and stem, whereas powdery mildew (*Golovinomyces ambrosiae*) and *Botrytis cinerea* were shown to be prevalent pathogens on the leaves and inflorescences. Also, an NGS approach was successfully applied to reveal the presence of the hop latent viroid (HLVd) and a cannabis mitovirus (CasaMV1) on most of the analyzed samples. These findings demonstrated the effectiveness of these approaches in providing insight into the virome of hemp and cannabis crops. All these methods were validated by performing the assays over several cropping years (2020–2023) and on many different hemp and cannabis genotypes from different geographical regions.

Simultaneously with the spread of the cultivation of cannabis and industrial hemp, and the increasing workforce that participates in various operations within the cannabis and hemp industry, the number of cases of allergic sensitization recorded has also increased. In this context, Loblundo and coworkers (Contribution 7) used the tandem mass spectrometry approach to obtain the proteomic profile of four varieties belonging to chemotypes from I to IV and assess the distribution of *C. sativa* allergens across chemotypes. Proteomic analysis revealed the presence of *C. sativa* proteins belonging to 50 distinct allergen families. Most of these proteins were typical airway (eighteen) and food (thirteen) allergens, but also two contact allergens were identified. Allergens able to sensitize through multiple routes were also detected. These included three clinically relevant and validated allergens of *C. sativa*; namely, profilin (Can s 2), non-specific lipid transfer protein (Can s 3), and Bet v 1-homolog (Can s 5). The authors observed that most of these allergens were expressed in all chemotypes, with Can s 3 found at high levels in hemp.

Despite the discovery of several protein allergens in *C. sativa* plants, these fail to explain all cases of allergic sensitization like urticaria and angioedema that are frequently observed in recreational cannabis users or workers exposed to cannabis and hemp plants in their occupational settings. Indeed, only half of these cases presented an increase in the pro-allergic IgE against *C. sativa*-specific proteins, thus suggesting a role for other components of *C. sativa* plants [19]. In light of this, Inan and coworkers (Contribution 8) used a murine model of dermatitis to verify whether CBD and the sesquiterpene β-caryophyllene may act as contact sensitizers. The results obtained by the authors revealed that β-caryophyllene but not CBD was responsible for allergic contact dermatitis-like symptoms. In particular, the repeated topical application of β-caryophyllene onto mice skin induced an itch response and dermatitis, whereas the histopathological analysis of skin tissues revealed significant edema, desquamation, and epidermal thickening, as well as a loss of filaggrin, an epidermal protein involved in the maintenance of the skin barrier. Symptomatic individuals also presented the trafficking of CD11b+ immune cells and mast cells; an increase in the chemokines C5/5a, sICAM-1, and IL-1ra, which are all important in the generation of allergic inflammation, and a dose-dependent increase in the serum levels of the IgE.

Overall, the Special Issue “*Cannabis sativa*: from plants to humans” illustrates well the two sides of this crop, as a source of beneficial bioactive compounds and as a psychoactive drug associated with adverse health outcomes. In light of the numerous industrial applications and the high economic value of the end products derived from hemp and cannabis plants, future research must focus on agronomic techniques and breeding programs that can maximize the quality of their raw materials (e.g., fiber, biomass, and phytochemicals). Also, further studies are needed to better understand the molecular targets and signaling pathways of cannabinoids and other phytochemicals from *C. sativa* plants. This will help to fully realize the potential of this crop to improve human health and minimize its side effects.

## Figures and Tables

**Figure 1:**
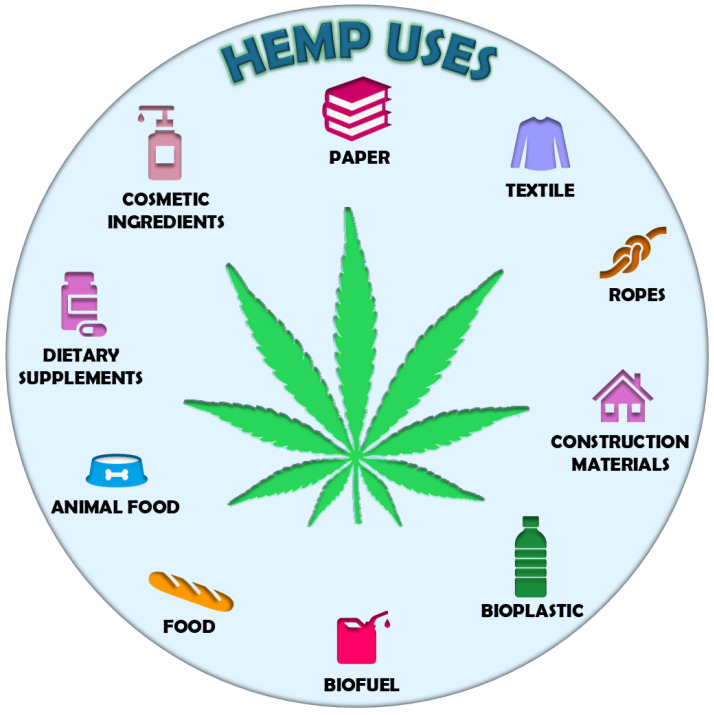
Schematic representation of the main industrial hemp applications.

**Figure 2:**
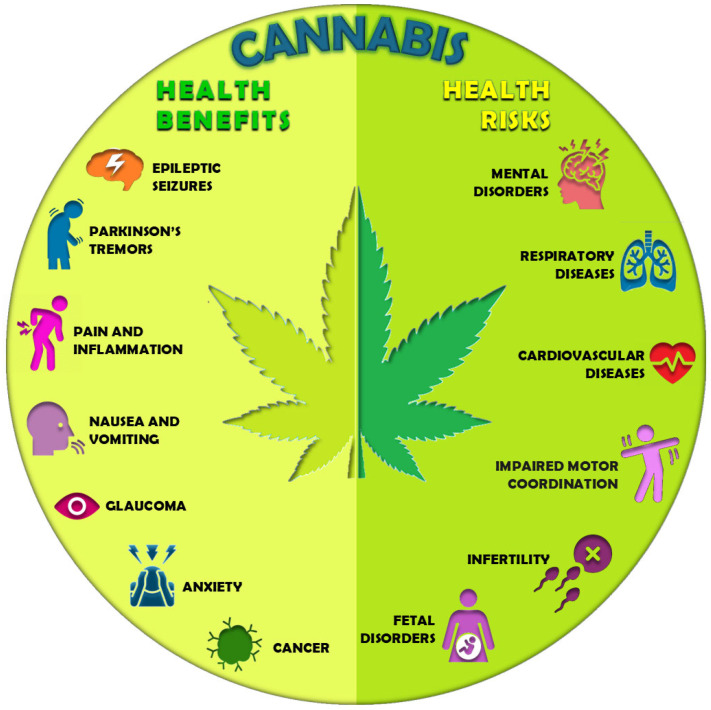
Therapeutic uses of cannabis-based products and negative effects of cannabis consumption for recreational use.
